# Graphene-Based Impregnation into Polymeric Coating for Corrosion Resistance

**DOI:** 10.3390/nano15070486

**Published:** 2025-03-24

**Authors:** Arti Yadav, Santosh Panjikar, R. K. Singh Raman

**Affiliations:** 1 Department of Chemical and Biological Engineering, Monash University, Melbourne, VIC 3800, Australia; arti.yadav@monash.edu; 2 Australian Nuclear Science and Technology Organisation, Australian Synchrotron, 800 Blackburn Road, Clayton, Melbourne, VIC 3168, Australia; santoshp@ansto.gov.au; 3 Department of Biochemistry and Molecular Biology, Monash University, Melbourne, VIC 3800, Australia; 4 Department of Mechanical and Aerospace Engineering, Monash University, Melbourne, VIC 3800, Australia

**Keywords:** graphene-based nano fillers, polymer-matrix composites, corrosion resistance

## Abstract

This review explores the development and application of the impregnation of graphene-based materials into polymeric coatings to enhance corrosion resistance. Derivatives of graphene, such as graphene oxide (GO) and reduced graphene oxide (rGO), have been increasingly integrated into polymer matrices to enhance polymers’ mechanical, thermal, and barrier properties. Various synthesis approaches, viz., electrochemical deposition, chemical reduction, and the incorporation of functionalised graphene derivatives, have been explored for improving the dispersion and stability of graphene within polymers. These graphene-impregnated coatings have shown promising results in improving corrosion resistance by enhancing impermeability to corrosive agents and reinforcing mechanical strength under corrosive conditions. While the addition of graphene notably enhances coating performance, challenges remain in achieving uniform graphene dispersion and addressing the trade-offs between thickness and flexibility. This review highlights current advancements, limitations, and future directions, with a particular emphasis on optimising the synthesis techniques to maximise corrosion resistance while maintaining coating durability and economic feasibility.

## 1. Introduction

Corrosion is destructive chemical/electrochemical process that causes great harm to the economy, as well as safety challenges worldwide. The annual losses due to corrosion are estimated at approximately USD 2.5 trillion, i.e., 3.4% of the global Gross Domestic Product (GDP) [1,2]. Although corrosion is an inevitable process (for fundamental thermodynamic reasons), it can be significantly mitigated using advanced corrosion-resistant materials, advanced material design, effective cathodic protection systems, cutting-edge corrosion inhibitors, and high-performance coatings.

In recent years, the use of various types of corrosion inhibitors, including inorganic, organic, and natural inhibitors, has gained significant attention due to their efficiency in mitigating corrosion. Natural inhibitors derived from plant extracts, in particular, have been highlighted for their eco-friendliness and efficiency. For instance, the study *“Investigating the Adsorption and Corrosion Protection Efficacy and Mechanism of Marjoram Extract on Mild Steel”* demonstrates the effectiveness of marjoram extract as a natural inhibitor through adsorption mechanisms [3]. Similarly, the study *“Electrochemical, Structural and Thermodynamic Investigations of Methanolic Parsley Extract as a Green Corrosion Inhibitor for C37 Steel”* underscores the potential of green inhibitors to provide sustainable corrosion protection [4]. Furthermore, the study *“Experimental and DFT Atomistic Insights into the Mechanism of Corrosion Protection of Low-Carbon Steel in an Acidic Medium by Polymethoxyflavones from Citrus”* offers a molecular-level understanding of the inhibition mechanisms provided by natural compounds, highlighting the role of flavonoids in enhancing adsorption and forming protective layers on metal surfaces [5]. These studies collectively emphasise the growing interest in eco-friendly corrosion inhibition strategies.

Though most means of ameliorating/mitigating corrosion may have been exhausted because of the age-old nature of corrosion and its huge economic implications, there have been recent advances that include the application of novel materials, such as graphene, for corrosion resistance [6,7]. In fact, the global market for graphene-based coatings is projected to grow significantly, reaching approximately USD 1 billion by 2028 [8], driven by a steady compound annual growth rate (CAGR) and demand across automotive, aerospace, and electronics applications.

In recent years, there has been a growing interest in eco-friendly corrosion inhibition approaches, particularly those involving graphene and biopolymer-based nanocomposites. As highlighted in the book *“Corrosion Protection of Metals and Alloys Using Graphene and Biopolymer-Based Nanocomposites”*, these materials offer significant advantages due to their non-toxic nature and exceptional barrier properties. The integration of biopolymers, such as chitosan, cellulose, and polyaniline, with graphene derivatives not only enhances the mechanical and anticorrosive properties of the coatings but also aligns with the increasing demand for sustainable and environmentally friendly corrosion protection solutions. This approach leverages the excellent impermeability of graphene to inhibit the ingress of corrosive species, while biopolymers contribute to enhanced adhesion and flexibility of the coating.

In recent years, the incorporation of graphene and its derivatives, such as graphene oxide (GO) and reduced graphene oxide (rGO), into polymer matrices has led to effective anticorrosion coatings that perform exceptionally even in aggressive corrosive conditions [9,10,11,12,13,14,15,16,17,18,19,20,21,22]. Incorporation of suitable derivatives of GO has been found to be particularly effective because the two-dimensional sheets of GO constitute a tortuous path for the inward transport of the corrosive species. Underpinned by this mechanism, Huang et al. [9] demonstrated that a nanocomposite coating of polyaniline (PANI) and GO nanosheets functionalised with polydopamine (PDA) considerably retarded the corrosion resistance of steel. Pu et al. [23] developed a composite coating of functionalised GO and a waterborne polyurethane (WPU) matrix, which forms a robust interpenetrating network, enhancing both hydrophobicity and adhesion to metal surfaces, and this resulted in an impressive 98% decrease in corrosion rate. Kumari et al. [24] showed that the incorporation of reduced GO (rGO) into a poly (4-vinylpyridine-co-butyl methacrylate) (PVPBM) matrix enhanced the corrosion protection efficiency to 95.4% while attaining high adhesion strengths. A study on GO–chitosan–silver composites demonstrated two orders of magnitude improvement in corrosion resistance [25]. Furthermore, the composites also enhanced thermal stability [21]. However, incorporation of GO into a polymeric matrix often raises the challenge of agglomeration of GO. Effective dispersion and compatibility of GO within the resin matrix are typically achieved through suitable covalent and non-covalent functionalisation of GO, such as through the incorporation of noble metals (e.g., Pt, Pd) or a semiconductor oxide (e.g., TiO_2_, ZnO), as well as chemical bonding with the two primary sites on GO/rGO sheets, i.e., oxygenated groups (–OH, –COC, and –COOH) for condensation and esterification, and carbon double-bond (C=C) sites on the basal plane for free-radical bonding [26]. Such functionalisation facilitates bonding with the polymer matrix, further enhancing GO’s ionic barrier properties [27]. Collectively, these studies underscore the versatility of the incorporation of graphene and GO-based coatings for varied industrial applications where both high corrosion resistance due to coatings and mechanical durability are of paramount importance [11,28].

**Figure 1:**
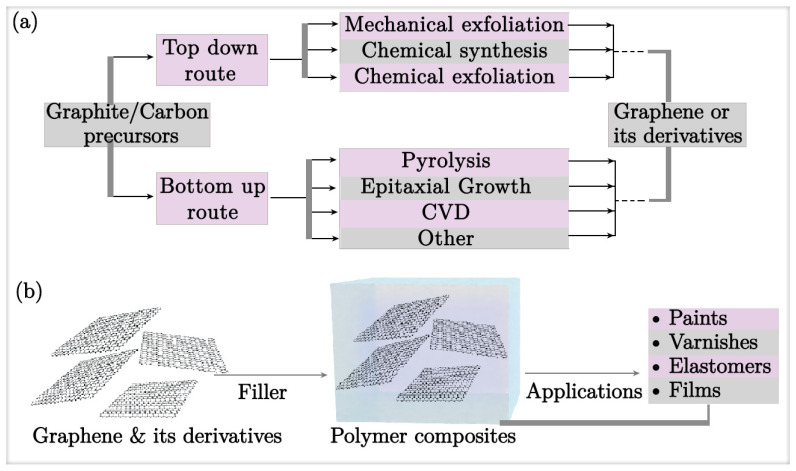
Schematic representation of (**a**) synthesis of graphene, (**b**) graphene and its derivatives as fillers in polymer composites.

This review discusses the incorporation of graphene and its derivatives into polymer composites as a cutting-edge approach to enhancing corrosion resistance. Figure 1 presents a schematic overview of the synthesis of graphene and its derivatives as fillers in polymer composites. By leveraging the excellent properties of graphene oxide (GO) and reduced graphene oxide (rGO), such as a high surface area, mechanical robustness, and impermeability, these composites considerably lengthen the transport pathways for corrosives within the coating, effectively and considerably retarding corrosion rates, making them highly attractive for industrial applications.

## 2. Preparation of Graphene and Its Derivatives

**Figure 2:**
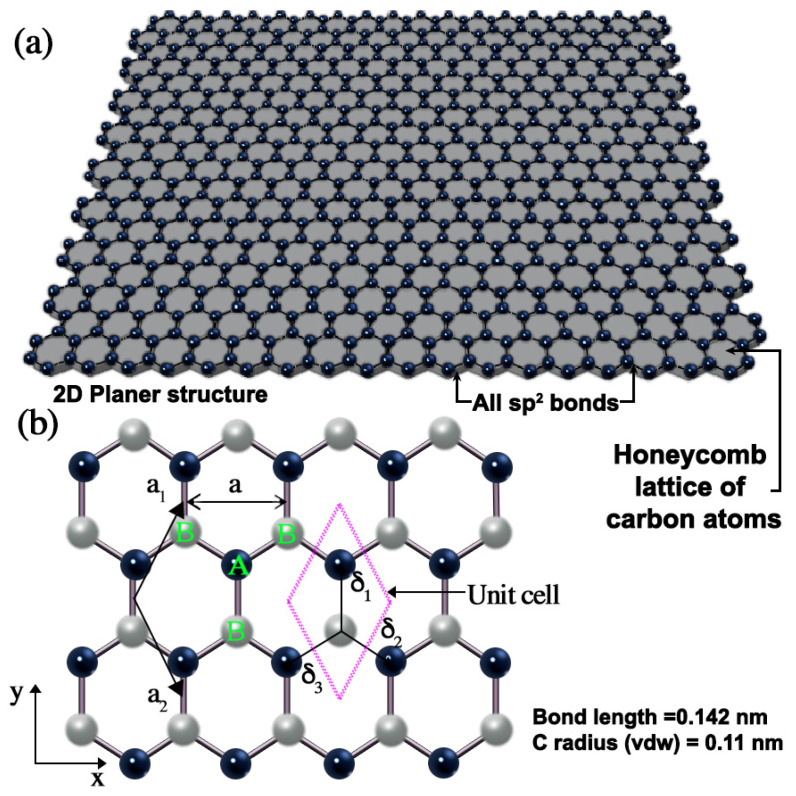
(**a**) Graphene lattice structure. (**b**) The honeycomb lattice configuration of monolayer graphene, where the grey and black circles represent carbon atoms located at specific lattice sites. Each atom in sublattice A (shown in green) has three nearest neighbours in sublattice B (also shown in green) and vice versa.

Graphene demonstrates a unique combination of properties essential for an ideal coating material, i.e., outstanding impermeability, pronounced chemical inertness, and exceptional mechanical toughness; no other single material possesses these attributes simultaneously [29,30,31,32]. Graphene is a two-dimensional (2D) sheet of sp^2^-hybridised carbon atoms arranged in a hexagonal lattice, as shown in Figure 2. This structure confers graphene with remarkable properties, making it the most investigated and attractive materials in recent years. Its exceptional characteristics include a remarkable electron mobility (2.5 × 10^5^ cm^2^V^−1^s^−1^), outstanding thermal conductivity (3000 WmK^−1^), excellent mechanical properties (Young’s modulus of 1 TPa), remarkable chemical stability, very high specific surface area (<2600 m^2^g^−1^), and exceptional optical transparency (97.4% transmittance at 550 nm) [33,34,35,36]. These properties can be tuned by adjusting factors such as the defect density, porosity, and number of graphene layers [33,37].

The π–π stacking interactions and strong van der Waals forces between graphene layers contribute to a tightly packed structure, which can enhance the barrier properties of graphene-based coatings. When graphene sheets aggregate, they form a dense layer that can effectively block the passage of corrosive agents like water, oxygen, and chloride ions, thereby protecting the underlying material from corrosion.

The synthesis of graphene includes mechanical exfoliation, epitaxial growth, liquid-phase exfoliation, electrochemical exfoliation, chemical oxidation and reduction processes, and chemical vapour deposition (CVD) [38,39,40,41,42,43,44,45,46,47]. The epitaxial growth of graphene on suitable substrates, such as silicon carbide (SiC), requires precise thermal control [46,47]. Liquid-phase exfoliation utilises specific solvents capable of dispersing and exfoliating individual layers, from graphite, thereby resulting in suspensions of graphene flakes within the solvent [48]. In chemical synthesis, graphite undergoes oxidation into graphene oxide (GO) flakes [49], which are subsequently chemically reduced to produce reduced graphene oxide (rGO) [45]. However, the graphene obtained through this method is not entirely pristine and contains numerous functional groups, which is why it is frequently designated as rGO, rather than graphene.

Electrodeposition of rGO has emerged as a promising technique due to its ability to provide precise control over the thickness of the deposited films and ensure better adherence and stability on metal substrates. This technique not only facilitates a uniform deposition but also enables the incorporation of functional materials such as nanoparticles to enhance the properties of rGO. For instance, the study *“Gold Nanoparticles Decorated Graphene as a High Performance Sensor for Determination of Trace Hydrazine Levels”* demonstrates the effectiveness of electrodeposition in achieving stable and uniform coatings with improved functional properties [50]. The enhanced adherence and stability provided by electrodeposition make it particularly suitable for applications requiring durable and high-performance anticorrosion coatings.

The chemical vapour deposition (CVD) technique for graphene synthesis entails the regulated generation of carbon atoms via catalytic dissociation of a hydrocarbon precursor on a substrate; the carbon atoms thus generated during this process self-organise on the substrate to form graphene [38,51,52]. Additionally, alternative methodologies such as spray coating, electrophoretic deposition, chemical conversion coating, flame spraying, electrochemical deposition, and laser-assisted transformation have also been explored for the synthesis of graphene.

CVD enables the production of high-quality, pristine graphene, rendering it the most prevalently utilised technique when highest-quality graphene is required [53,54,55,56]. CVD-produced graphene films are characterised by minimal defects and high purity. However, the substrates used for CVD graphene must be capable of catalytically decomposing the hydrocarbon precursor to generate nascent carbon atoms, as depicted in [51].

### 2.1. Preparation Methods of Graphene and Its Derivatives for Polymer Composites

**Figure 3:**
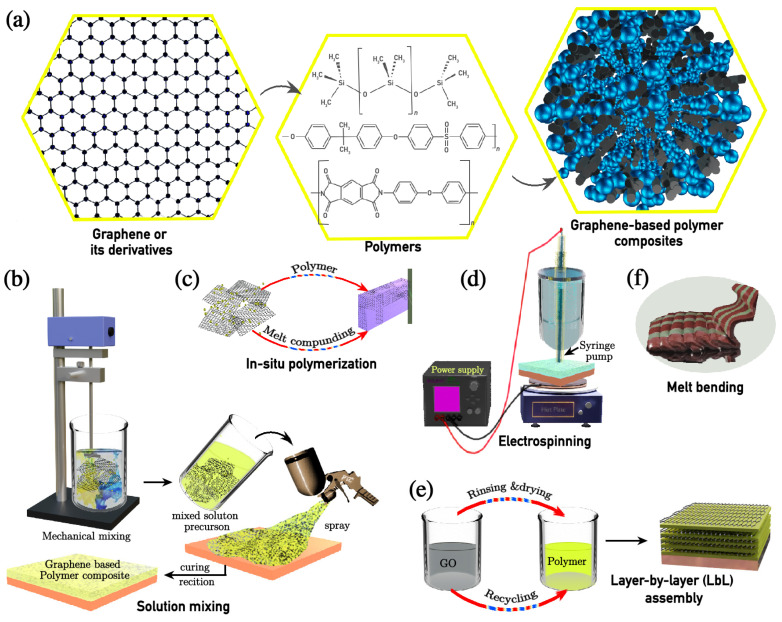
(**a**) Schematic illustration of a polymer composite with impregnated graphene or its derivatives. This illustrates various widely employed preparation methods of graphene and its derivatives for polymer composites: (**b**) solution mixing, (**c**) in situ polymerisation, (**d**) electrochemical reaction, (**e**) layer-by-layer (LbL) assembly, and (**f**) melt blending.

The preparation methodologies significantly influence the composite structure, dispersion quality, and resultant properties. Common methods for preparing graphene-based polymer composites (Figure 3b–f) include solution mixing, in situ polymerisation, electrochemical techniques (electrospinning and electrodeposition), layer-by-layer (LbL) assembly, and melt blending. These techniques offer various advantages in terms of dispersion control, structural precision, scalability, and environmental considerations.

#### 2.1.1. Solution Mixing

Solution mixing is a widely utilised technique for synthesising graphene and its derivative-based polymer composites due to its simplicity and efficiency. The process involves dispersing graphene or its derivatives (e.g., graphene oxide, reduced graphene oxide, functionalised graphene) in an appropriate solvent such as N,N-dimethylformamide (DMF), tetrahydrofuran (THF), chloroform, or ethanol through simple mixing, sheer/mechanical stirring, or ultrasonication to ensure uniform exfoliation (Figure 3b). The polymer is then introduced, followed by solvent removal via evaporation or vacuum distillation to obtain the final composite [57]. The quality of dispersion and interfacial interactions strongly influence the resulting properties. To enhance these interactions, surface functionalisation strategies such as polymer grafting or surfactant-assisted dispersion can be employed [13].

#### 2.1.2. In Situ Polymerisation

In situ polymerisation ensures a uniform dispersion of graphene and its derivatives by initiating polymerisation in the presence of graphene sheets, allowing polymer chains to grow around the sheets, thereby preventing agglomeration (Figure 3c) [57,58,59]. The process starts with the dispersion of graphene in a monomer solution, followed by polymerisation using thermal, chemical, or photoinitiators. Controlled polymerisation methods such as atom transfer radical polymerisation (ATRP) and reversible addition–fragmentation chain transfer (RAFT) polymerisation allow molecular weight control and improved compatibility between graphene derivatives and the polymer matrix [13,60].

#### 2.1.3. Electrochemical Reaction

Electrospinning is a scalable method for fabricating graphene and its derivatives with a high aspect ratio for polymer-nanofibre composites. The process employs a high-voltage (5–20 kV) electric field to create a charged polymer jet that elongates and solidifies into nanoscale fibres, as illustrated in Figure 3d [61,62]. Common solvents used in electrospinning include DMF, acetone, and chloroform, which help control polymer chain entanglement and jet stability. Advanced electrospinning techniques, such as coaxial electrospinning and emulsion electrospinning, enable the fabrication of core–shell and multi-layered nanofibres with tailored functionalities [63].

Electrodeposition is an efficient and precise technique for fabricating nanocomposites via electrochemical processes. This method facilitates the electro-polymerisation of a graphene/polymer nanocomposite from a monomer, dopant (if needed), and graphene oxide (GO), under controlled electrochemical conditions. The deposition occurs at a defined potential and ceases upon reaching the requisite charge transfer threshold [62]. As a result, the synthesised nanocomposite forms a uniform and adherent coating on the electrode surface.

#### 2.1.4. Layer-by-Layer (LbL) Assembly

The LbL assembly technique is a highly controlled synthesis method for constructing multilayered graphene and its derivatives for polymer-fibre nanocomposites. The process involves sequential deposition of graphene-based materials and polymeric layers, utilising electrostatic interactions, hydrogen bonding, or van der Waals forces (Figure 3e) [64]. This method enables precise tuning of the thickness, surface chemistry, and composite properties. The stability and charge compatibility of graphene suspensions are crucial for achieving uniform film growth. Common solvents used in LbL assembly include water, ethanol, and methanol, ensuring the stable dispersion of graphene nanosheets [60].

#### 2.1.5. Melt Blending

Melt blending is an industry-preferred, solvent-free synthesis method for fabricating graphene and its derivatives for polymer-fibre nanocomposites. The technique involves dispersing graphene into a molten polymer matrix under high shear forces, typically using twin-screw extrusion or injection moulding (Figure 3f) [62]. The effectiveness of melt blending depends on graphene’s dispersion quality, which is often enhanced by surface modifications or compatibilisers. While this method offers scalability and environmental advantages, achieving a uniform dispersion remains challenging due to the high viscosity of molten polymers. Melt blending is widely used for fabricating conductive polymer composites, structural reinforcements, and lightweight functional materials [65].

## 3. Incorporation of Graphene and Its Derivatives into Polymer Composites

Incorporation of graphene and its derivatives into polymer composites has emerged as a promising strategy for enhancing corrosion resistance (schematic illustration shown in Figure 3a). Suitably functionalised graphene nanoplatelets have seamlessly been integrated into a wide range of polymeric matrices, which include epoxy [66], polyaniline [67], polyurethane [68], polyamide [69], polystyrene [70], polymethylmethacrylate [71], polypropylene [72], polydopamine [73], polyetherimide [74], polyvinyl butyral [75], polyvinyl alcohol [76], polypyrrole [77], biopolymer [78], silane [79], and polydimethylsiloxane [80], among others. These composites enhance properties like durability, flexibility, and corrosion resistance.

For instance, polyaniline/graphene composites (PAGCs) have demonstrated remarkable barrier properties against oxygen and water, outperforming traditional materials like neat polyaniline or even polyaniline/clay composites [67,81]. This enhanced performance is primarily due to the well-dispersed graphene nanoplatelets that inhibit the transport of corrosive molecules, thereby enhancing the corrosion protection capabilities of the coatings [81]. Functional groups of graphene oxide (GO) can be further functionalised to facilitate bonding with the polymer matrix. Incorporation of functionalised GO into polystyrene remarkably improved corrosion protection efficiency (from 37.90% to 99.53%, with just 2 wt% of the additive) [70]. Likewise, graphene/pernigraniline composites (GPCs) enhanced corrosion protection of copper substrates. These composites not only mitigate the corrosion but can also enhance other properties of polyaniline [74,82]. The use of 4-aminobensoyl group-functionalised graphene-like sheets as a conductive filler in these composites enables improved dispersion within the polymer matrix, which is crucial for forming an effective barrier against corrosive agents [11]. However, this requires the specific functionalisation of GO for effective bonding with the polymer matrix, and the incorporation of properly functionalised GO can improve both mechanical and barrier properties of the coating [13]. For instance, a study on PSS-PANI/rGO composite found the ultimate tensile strength and toughness of the epoxy composite with just 0.5 wt% PSS-PANI/rGO to be improved by 39% and 127%, respectively, when compared to the respective values of the plain epoxy [83]. Suitably functionalised GO facilitates the formation of a network that enables improved properties across the coating. With impressively improved properties of functionalised GO-polymer composites, as discusses earlier, these advanced materials hold significant potential for practical applications in various industries, particularly in corrosive environments [84]. Table 1 outlines the advancements in graphene-based polymer composites, highlighting the role of various polymer matrices and fabrication techniques in the enhancement of corrosion, mechanical, and barrier properties.

The incorporation of few-layer graphene and graphene oxide (GO) as fillers in polymer composites has emerged as a promising strategy to enhance corrosion resistance [12]. Both graphene and GO serve to improve the barrier properties of these composites by effectively retarding the permeability of corrosive agents through the coating. Coatings of composites incorporated with platelets of functionalised GO produce a considerably superior corrosion resistance when compared to the composite coatings with other fillers [12]. This enhancement is attributed to the structure of graphene, which is an atomically thick 2D platelet with a remarkably high aspect ratio, which makes the path very tortuous for permeating corrosive agents, thereby providing a considerably more effective barrier against corrosion [19]. Besides providing an enhanced permeability barrier, the polymer-GO composite also possesses enhanced mechanical properties and overall durability. In this context, electroactive polyimide/graphene nanocomposite coatings are particularly attractive for anticorrosive properties. Such coatings are prepared through thermal imidisation to incorporate carboxyl-graphene nanosheets that significantly retard gas permeation, thereby enhancing corrosion resistance due to the coatings [28]. Further, the presence of amino-capped aniline trimer (ACAT) units within such composites is noted for their redox catalytic capabilities, which can induce the formation of passive metal oxide layers on the substrate, providing an additional layer of protection against corrosion [28].

### 3.1. Graphene/Polyaniline Composites

**Figure 4:**
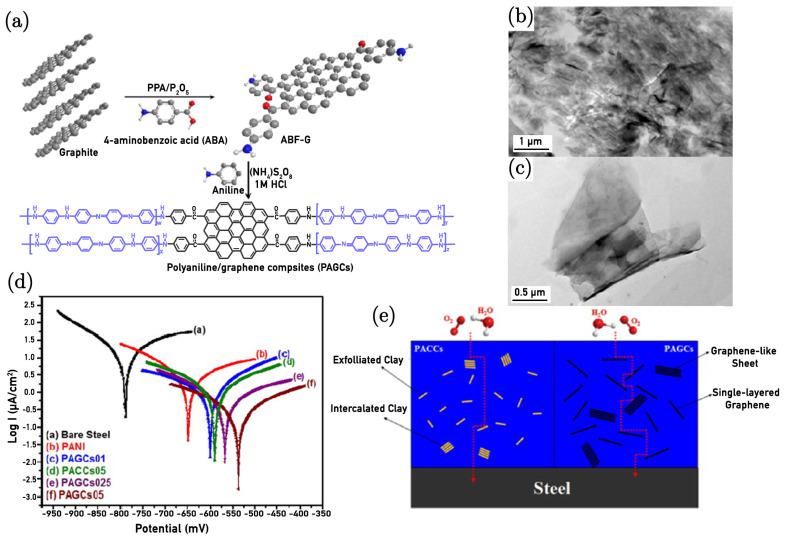
(**a**) Synthesis process for PANI/graphene composites (PAGCs). (**b**) TEM images of PAGCs05 at a low magnification and (**c**) high magnification. (**d**) Tafel plots comparing bare steel and PANI-coated, PAGCs01-coated, PACCs05-coated, PAGCs025-coated, and PAGCs05-coated electrodes, assessed in a 3.5 wt.% NaCl solution. (**e**) Conceptual illustration of O_2_ and H_2_O molecules traversing a tortuous path through PACCs and PAGCs [11].

Chang et al. [11] demonstrated very impressive anticorrosion properties due to coatings of polyaniline/graphene composites (PAGCs). The PAGC coatings considerably reduce the permeability of gases such as oxygen (O_2_) and water vapour (H_2_O) compared to traditional polyaniline and polyaniline/clay composites (PACCs). Specifically, PAGCs with 0.1 wt.% ABF-G loading showed a reduction by approximately 55% and 60% in O_2_ and H_2_O permeabilities, respectively. This improvement is attributed to the high aspect ratio of the 4-aminobenzoyl group-functionalised graphene-like sheets (ABF-Gs) and a high aspect ratio of approximately 500 (compared to 220 for clay), which presents a more tortuous path for gas molecules, effectively enhancing the barrier properties of the composites. The study further reveals that increasing the ABF-G loading results in even greater enhancements in barrier properties, making PAGCs more effective gas barrier materials for anticorrosion coatings than PACCs. Figure 4a illustrates the synthesis process schematic for PANI/graphene composites (PAGCs), demonstrating the structural and functional characteristics of the composites, and the TEM images (Figure 4c,d) confirm that ABF-G is well-dispersed within the polyaniline matrix, indicating good compatibility and effective functionalisation. These structural modifications and the presence of nitrogen from the functional groups contribute to the enhanced barrier properties and electrical conductivity of PAGCs.

Another study on GO/polyaniline composites by Gao et al. [86] investigated the enhancement of corrosion resistance due to waterborne epoxy coatings developed upon the incorporation of polyaniline (PANI) nanorods and nitrogen and fluorine dual-doped graphene oxide (NFGO) composites. XRD confirmed the crystalline structure of PANI and the integration of NFGO. TEM images reveal that PANI forms short rod-like structures with diameters of approximately 200−400 nm. When combined with NFGO, PANI rods grow on the graphene surface, increasing their aspect ratio due to additional nucleation sites provided by the graphene. They identified the morphology of PANI to be significantly influenced by the polymerisation time, which is crucial for the coating’s performance. A shorter than optimal polymerisation time results in a coral-like particulate structure, while an excessively long time leads to agglomeration and an irregular morphology. The optimal polymerisation time results in a rod-like structure, which is essential for effective dispersion and interaction with nitrogen and fluorine dual-doped graphene oxide (NFGO). Electrochemical impedance spectroscopy (EIS) of PANI/NFGO/WEP coatings conferred the highest corrosion resistance, with R_ct_ values considerably higher than that for neat PANI coating. The salt spray test results show that PANI/NFGO/WEP coatings effectively inhibit the penetration of corrosive media, maintaining good adhesion even under wet conditions. This is attributed again to the two-dimensional GO blocking water molecule penetration, as a result of the effective bonding of GO with the polymeric matrix. The coated substrate demonstrated minimal rusting, even after extended exposure, highlighting its durability.

The enhanced corrosion resistance observed in the coatings can be attributed to the synergistic effect of polyaniline (PANI) and nitrogen and fluorine dual-doped graphene oxide (NFGO). The dual-doping of NFGO with nitrogen and fluorine not only improves electrical conductivity but also introduces additional active sites for the polymerisation of PANI. This facilitates a more uniform dispersion and stronger interaction between PANI and NFGO within the coating matrix. The high specific surface area and excellent conductivity of NFGO, combined with the passivation effect of PANI, create a robust barrier against the ingress of corrosive media.

In addition to the barrier protection mechanism, the incorporation of PANI provides anodic protection by forming a passivation layer on the substrate surface. This layer can shift the corrosion potential to more noble values, thereby reducing the anodic dissolution of the metal substrate. PANI, being a conductive polymer, also enables electron transfer processes that support the formation of passive oxide layers, further enhancing the protective capabilities of the coating. This dual protection mechanism—barrier and anodic protection—significantly improves the corrosion resistance compared to traditional polymer/clay composites.

**Figure 5:**
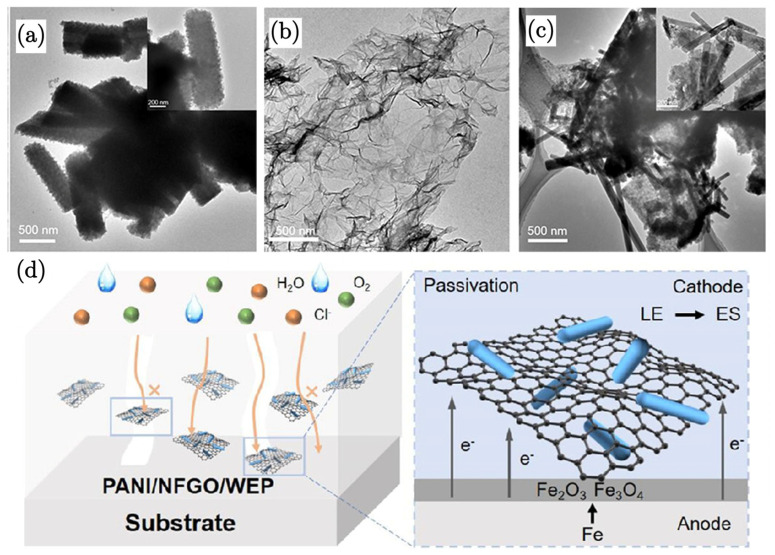
(**a**) TEM image of PANI, (**b**) TEM image of NFGO, and (**c**) TEM image of the PANI/NFGO composite. (**d**) Schematic illustration of the corrosion protection mechanism [86].

The importance of this dual protection mechanism is further demonstrated in Figure 5d, which shows a substantial reduction in corrosion current density for the PANI/NFGO composite coatings compared to the control samples. The lower corrosion current density indicates a more effective suppression of electrochemical reactions at the metal surface, corroborating the protective role of both PANI and NFGO.

Both studies [11,86] underscore the potential of polyaniline/graphene composites as advanced anticorrosion coatings. The combination of a more tortuous path for diffusing gas molecules and the formation of a passive protective layer effectively prevent corrosion, making these composites a promising alternative to traditional coatings.

### 3.2. Graphene Oxide–Polymer Bilayer Coating

**Figure 6:**
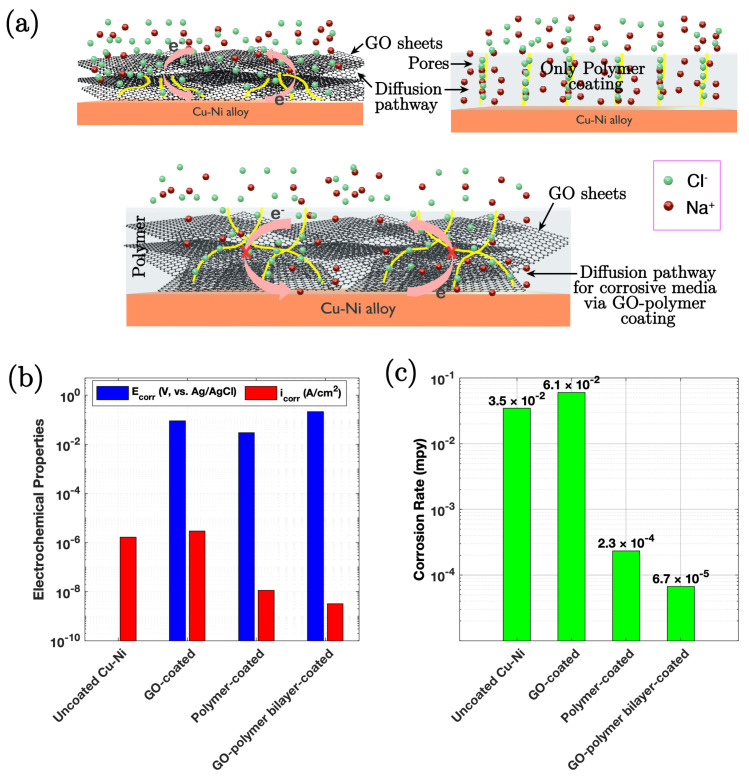
(**a**) Schematic representation of the corrosion protection mechanism for GO-coated, polymer-coated, and GO–polymer bilayer-coated Cu-Ni samples. (**b**) Bar chart depicting *E_corr_* and *i_corr_* values of the uncoated, GO-coated, polymer-coated, and GO–polymer bilayer-coated Cu-Ni samples in a 3.5% (*w*/*v*) NaCl solution after 1 h of stabilisation. (**c**) Corrosion rates of uncoated, GO-coated, polymer-coated, and GO–polymer bilayer-coated Cu-Ni samples in a 3.5% (*w*/*v*) NaCl solution, in mils per year [85].

George et al. [85] studied a robust bilayer coating of graphene oxide (GO) and acrylic polymer on Cu-Ni alloys, for corrosion resistance in chloride environments. GO was prepared using a modified Hummers’ method and deposited onto the alloy via electrophoretic deposition (EPD) at a constant voltage of 10 V for 60 s, followed by overnight drying at room temperature. The acrylic polymer layer was applied using a dip coating technique, where the GO-coated samples were immersed in a 10% PMMA solution in THF for 30 s, followed by lifting at a controlled speed. The process was repeated twice, and the samples were cured at 110 °C for 12 h. In their study, the surface characteristics of the GO-coated Cu-Ni alloy, the polymer-coated alloy, and the GO–polymer bilayer-coated alloy were examined by AFM. The GO-polymer bilayer coating was found to be 5–6 µm thick, which provided a robust barrier against corrosion, with a protection efficiency of 99.8%, attributed to the synergistic properties of GO and the polymer. As evidenced by the EIS results (Figure 6b), the bilayer coating shows a significantly higher coating resistance compared to the uncoated and single-layer coated samples, confirming the superior protective nature of the former in aggressive chloride environments. The GO layer enhances impermeability, while the polymer provides good film-forming, insulating, and adhesion properties.

### 3.3. Graphene–Epoxy Powder Coating

**Figure 7:**
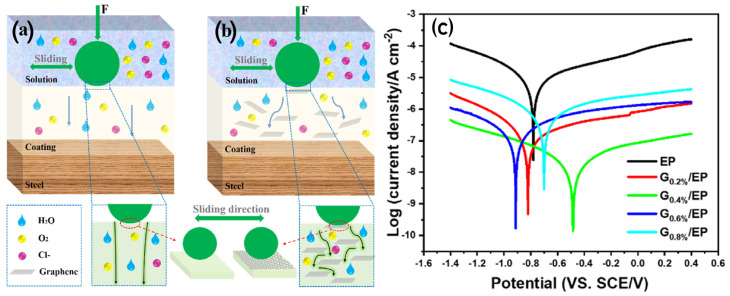
(**a**) Schematic illustration of the wear improvement mechanism and (**b**) corrosion resistance mechanism in the G/EP composite powder coating. (**c**) Potentiodynamic polarisation curves of the neat EP and G/EP composite coatings [87].

Zhang et al. [87] investigated the impact of graphene nanosheet addition to epoxy powder coatings on the wear and corrosion resistance. A schematic illustration of the wear improvement mechanism and corrosion resistance mechanism in the G/EP composite powder coating are given in Figure 7. The addition of 0.4 wt% graphene nanosheets significantly enhanced the coating properties. The graphene nanosheets enhanced lubrication of the wearing surface, thereby reducing wear. In addition, the dense structure of the nanosheet-containing surface improved adhesion, which suppressed deformation and microcracks, resulting in a shallow and narrow wear track, beneficial for wear resistance.

In terms of corrosion resistance, the incorporation of graphene nanosheets increases the compactness of the coating, which retards transport of the corrosive medium. This results in an excellent improvement in corrosion protection (Figure 7c), as evident from the high |Z| 0.01 Hz value of 9.65 × 10^5^ Ω cm^2^ after 50 days of immersion. The R_c_ and R_ct_ values, which reflect the physical barrier effect and severity of the corrosion reaction, respectively, also suggest that the G 0.4%/EP sample maintains the highest values, demonstrating enhanced corrosion resistance.

A previous study [78] also highlights the considerably inferior performance of epoxy powder coating without graphene nanosheets. The loose structure, inferior adhesion, and lack of a lubricating phase leave the coating prone to delamination or peeling during wear, leading to coating failure. Surface defects such as pinholes, voids, and microcracks can extend below the sliding surface, forming channels for corrosive medium penetration, which accelerates damage and coating peeling. The graphene-reinforced coating, however, mitigates these problems, as described earlier.

Overall, the findings suggest that the impregnation of graphene nanoplates into coatings offers a promising strategy for providing robust resistance to degradation due to combined corrosion and mechanically assisted degradation, such as corrosion–wear.

### 3.4. GO Modified with Polyamide–Epoxy/Zn Coating

**Figure 8:**
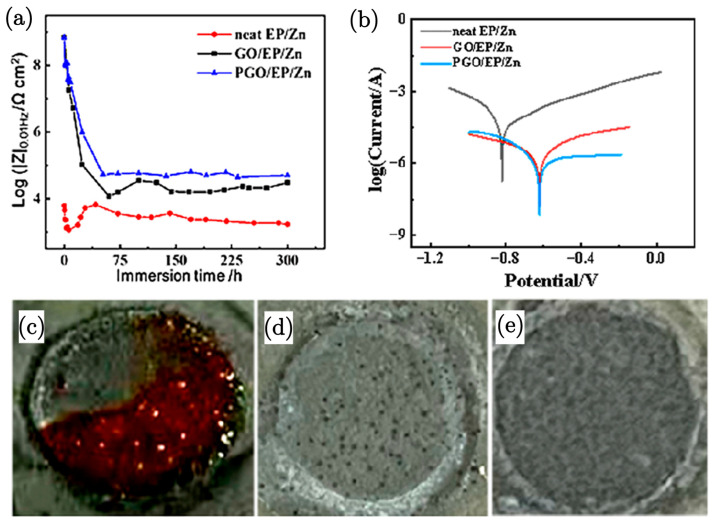
(**a**) Impedance dependence of neat EP/Zn, GO/EP/Zn, and PGO/EP/Zn coatings over immersion time in a 3.5 wt.% NaCl solution. (**b**) Tafel curves of neat EP/Zn, GO/EP/Zn, and PGO/EP/Zn samples after 300 h of immersion in 3.5 wt.% NaCl solution. Visual observations of coating samples exposed to 3.5 wt.% NaCl solution after 300 h of immersion: (**c**) neat EP/Zn, (**d**) GO/EP/Zn, and (**e**) PGO/EP/Zn, with a circle diameter of 1.1 cm in each image [88].

Zn-rich polymer composite paint is a widely applied type of coating due to the effect of the Zn particles, which work as anodes to provide cathodic protection. In order to enhance the anticorrosion effect of epoxy/zinc (EP/Zn) coatings, graphene oxide (GO) can be added as filler. A recent study [88] showed GO modified with polyamide to further enhance the corrosion resistance on a carbon steel (Q235) substrate. A comprehensive study demonstrated an enhanced corrosion resistance of epoxy–zinc (EP/Zn) coatings upon the addition of graphene oxide (GO) and its modified form, polyamide-modified GO PGO [88]. The incorporation of 0.3 wt.% GO into the EP/Zn coating significantly improved corrosion resistance, as indicated by the increase in the R_p_ to 13,115 Ω cm^2^ for PGO/EP/Zn, compared to 5386 Ω cm^2^ for GO/EP/Zn. This enhancement is attributed to the grafting of polyamide curing agent (PA-651) onto PGO, which improves its dispersibility and interaction with the epoxy matrix, effectively plugging micropores and preventing the permeation of corrosive agents. The study also reported that the R_t_, a critical parameter for evaluating corrosion resistance, was 64,520 Ω cm^2^ for PGO/EP/Zn, i.e., more than double that of GO/EP/Zn at 31,830 Ω cm^2^. This significant increase in R_t_ suggests a lower rate of electrochemical corrosion, indicating the superior protective properties of the PGO/EP/Zn coating. The enhanced performance is further supported by the visual evidence of the absence of red ferric compound on the surface of the GO/EP/Zn and PGO/EP/Zn coatings after immersion tests, i.e., unlike the neat EP/Zn coating (Figure 8), which showed extensive red corrosion products.

The mechanism behind the improved performance described above is linked to the modification of GO with PA-651. This modification results in a reduced O/C atomic ratio, indicating a decrease in oxygen-containing functional groups and an increase in re-aromatisation, which enhances the dispersion stability of PGO in organic solvents. This was confirmed by Raman spectroscopy, which revealed shifts in the D and G bands, suggesting charge transfer between the polyamide and GO, contributing to the improved properties of the composite.

In summary, the study effectively demonstrates that the in situ modification of GO with a curing agent significantly enhances the corrosion resistance of epoxy–zinc coatings. The findings highlight the potential of using modified GO in protective coatings to extend the lifespan of metal substrates in corrosive environments.

### 3.5. Graphene/Polyeugenol Composite

**Figure 9:**
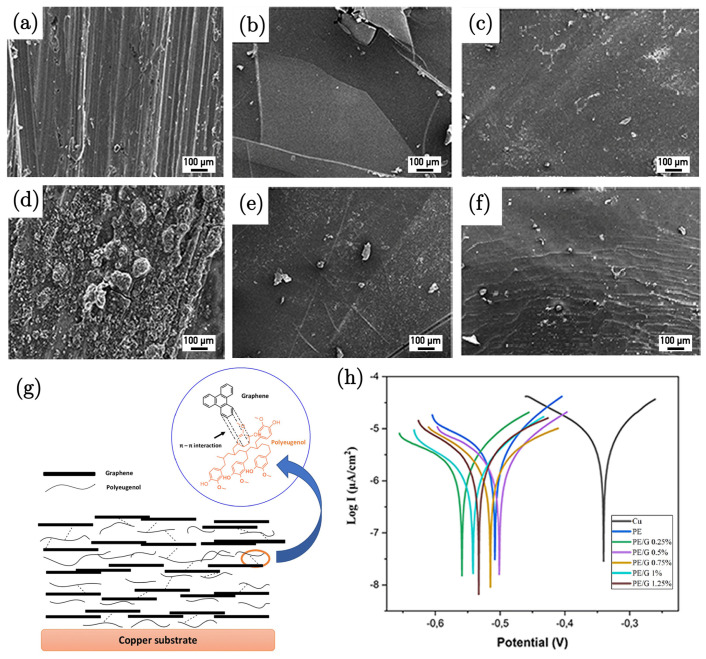
SEM images of bare Cu (**a**) before and (**d**) after immersion, PE-coated Cu (**b**) before and (**e**) after immersion, and PE/G 1.25%-coated Cu (**c**) before and (**f**) after immersion. (**g**) Schematic illustration of the PE/G composite coating on copper. (**h**) Tafel plots of Cu, PE, PE/G 0.25%, PE/G 0.5%, PE/G 0.75%, and PE/G 1% in 3.5 wt.% NaCl aqueous solution [89].

Adiwibawa Prasetya et al. [89] investigated the anticorrosion properties of a polyeugenol/graphene (PE/G) composite coating on copper metal. The results demonstrate that the addition of graphene significantly enhances the corrosion resistance of the coating. The electrochemical analysis shows a decrease in the corrosion rate with an increasing graphene content, as indicated by a reduction in the corrosion current density (I_corr_), which is attributed to graphene acting as a physical barrier, preventing/hindering corrosive agents like oxygen and chloride ions from reaching the metal surface. The potentiodynamic polarisation revealed that the PE/G composite offers superior anticorrosion properties compared to pure polyeugenol. The corrosion protection efficiency increased from 37% to 78% with the incorporation of 1.25 wt% of graphene into the polyeugenol matrix (Figure 9h). This improvement highlights the effectiveness of graphene as a reinforcing agent in polymer matrices for corrosion protection. Morphological analysis using SEM before and after immersion (Figure 9a–f) further supported these findings. The mechanism underlying the enhanced corrosion resistance involves the formation of a physical barrier by graphene (Figure 9g). This is supported by the absence of corrosion products in the XRD analysis of the coated samples, indicating that the PE/G coating effectively prevents the formation of corrosion products such as Cu_2_O and Cu_2_(OH)_3_Cl, which were observed in the case of the uncoated samples. The presence of graphene in the composite makes it challenging for corrosive species to penetrate the coating, thereby enhancing its protective capabilities.

SEM of the morphology also suggested that the PE/G-coated metal exhibited higher hydrophobicity compared to uncoated or PE-coated metal. This increased hydrophobicity contributes to the coating’s ability to repel water and other corrosive agents, further enhancing its anticorrosion performance. Overall, the study provides strong evidence for the potential of PE/G composites as effective anticorrosion coatings, with significant potential for industrial applications.

### 3.6. Poly(m-Phenylenediamine)-Encapsulated Graphene

**Figure 10:**
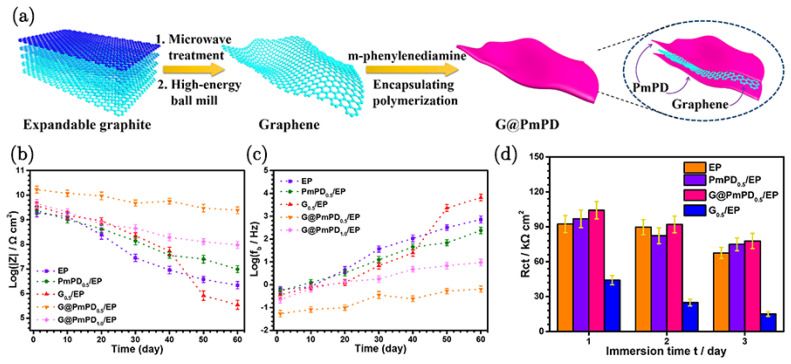
(**a**) Preparation process of G@PmPD. (**b**) ∣*Z*∣_10m Hz_ and (**c**) *f*_b_ values for EP, PmPD_0.5_/EP, G_0.5_/EP, G@PmPD_0.5_/EP, and G@PmPD_1.0_/EP coatings as a function of immersion time. (**d**) Fitting results of *R*_ct_ for different scratched samples [90].

Zhang et al. [90] used a two-step process to prepare poly(m-phenylenediamine)-encapsulated graphene (G@PmPD); the preparation method is as illustrated in Figure 10a. High-energy ball milling provided impermeable graphene-encapsulated poly-m-phenylenediamine (via non-covalent π-π interactions), which reduced the conductivity to 1.2 × 10^−7^ S cm−^1^ and mitigated/ameliorated the electrochemical corrosion-promoting effect due to the cathodic nature of graphene. The amino-rich surface of G@PmPD improved dispersibility and compatibility with organic solvents and polymer matrices, facilitating the application of the composite coatings, thereby improving corrosion resistance. The incorporation of 0.5 wt% of G@PmPD into an epoxy matrix coating markedly improved the corrosion protection of steel substrates in a 60-day test. This enhancement is attributed to the impermeability of G@PmPD as a result of the enhanced compactness due to cross-linking between G@PmPD and the epoxy resin. Notably, even when the composite coating is damaged, the presence of G@PmPD effectively mitigates the damage (Figure 10b–d).

The morphological images revealed that the encapsulation of graphene by PmPD results in a thicker and less transparent structure compared to the original graphene. Elemental mapping by EDS confirms the uniform distribution of nitrogen from PmPD on the G@PmPD surface, verifying successful encapsulation, which contributes to the improved properties of the composite coating. The mechanism of enhanced corrosion protection involves several key factors. The insulating encapsulation of graphene by PmPD reduces its conductivity, thereby enhancing corrosion protection. The π-π interaction between PmPD and graphene ensures a stable and uniform dispersion within the epoxy matrix, which facilitates effective tortuosity of the diffusion path for corrosives, delaying their penetration. Additionally, the cross-linking between G@PmPD and the epoxy resin increases the compactness and cross-link density of the coating, further enhancing its barrier properties. This comprehensive approach also effectively addresses the challenges of graphene agglomeration, which is often a problem in the incorporation of graphene into a polymeric matrix.

### 3.7. GO-Polyethylene Imine-Polyacrylic Composite

**Figure 11:**
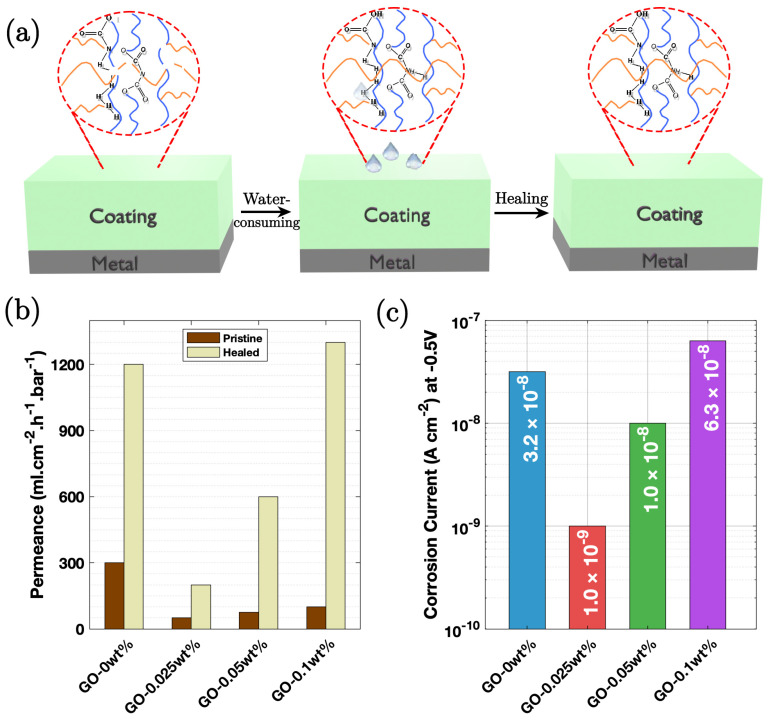
(**a**) Illustration of the self-repairing mechanism, where hydrogen bonds form upon water absorption, enabling healing. (**b**) Changes in water permeability for coatings in different states, highlighting the effect of the self-healing process. (**c**) EIS results of the PEI-PAA with GO-containing (GO-0 wt%, GO-0.025 wt%, GO-0.05 wt%, and GO-0.1 wt%) coatings exposed to air following 360 h of immersion in a 3.5 wt.% NaCl solution, demonstrating corrosion resistance [91].

Zheng et al. [91] prepared a graphene/supramolecular polymer (PEI-PAA-GO) based on a graphene oxide (GO) composite with branched poly (ethylene imine) (PEI) and poly (acrylic acid) (PAA). Mild steel substrate coupons with a proper surface finish and cleaning were coated with PEI-PAA-GO coating. Coatings with increasing GO contents were named GO-0 wt%, GO-0.025 wt%, GO-0.05 wt%, and GO-0.1 wt%. EIS results (Figure 11c) indicated that the GO-containing coatings (GO-0.05–GO-0.15 wt%) exhibited superior corrosion resistance than the one without GO (GO-0 wt%). The self-repairing performance of the coatings was assessed by measuring the healing efficiency (η healing). GO-0.025 wt% showed a higher healing efficiency of 143.75 Ω h^−1^ compared to 90 Ω h^−1^ for GO-0 wt%. The enhanced self-repairing ability of GO-containing coatings is attributed to stronger intramolecular hydrogen bonding and electrostatic interactions.

The mechanism of protection involves the combination of the physical barrier effect of the 2D lamellar graphene oxide and the water-retaining property of the supramolecular polymer. This synergistic effect resulted in high corrosion resistance, with an impedance modulus reaching 8.0 × 10^3^ Ω cm^2^, which is four times higher than that of a pure PEI/PAA coating. The Bode-phase diagrams suggested the degradation characteristics of certain coatings over time, while others maintained high/durable corrosion resistance. The water uptake too decreases significantly, preventing swelling and enhancing the protective capability of the coating. Figure 11a illustrates a schematic of the degradation and self-repairing mechanisms. The self-repairing is attributed to the re-establishment of the polymer network at the fracture surface, facilitated by hydrogen bonding.

An overview illustrating the corrosion resistance performance of different graphene-based polymer composites, highlighting their material compositions, corrosive environments, and critical electrochemical parameters such as inhibition efficiency, Tafel slopes (*βa/βc*), corrosion current density (*I_corr_*), and corrosion potential (*E_corr_*), is provided in Table 2.

## 4. Overview of Functionalised Graphene in Polymer Composites

**Figure 12:**
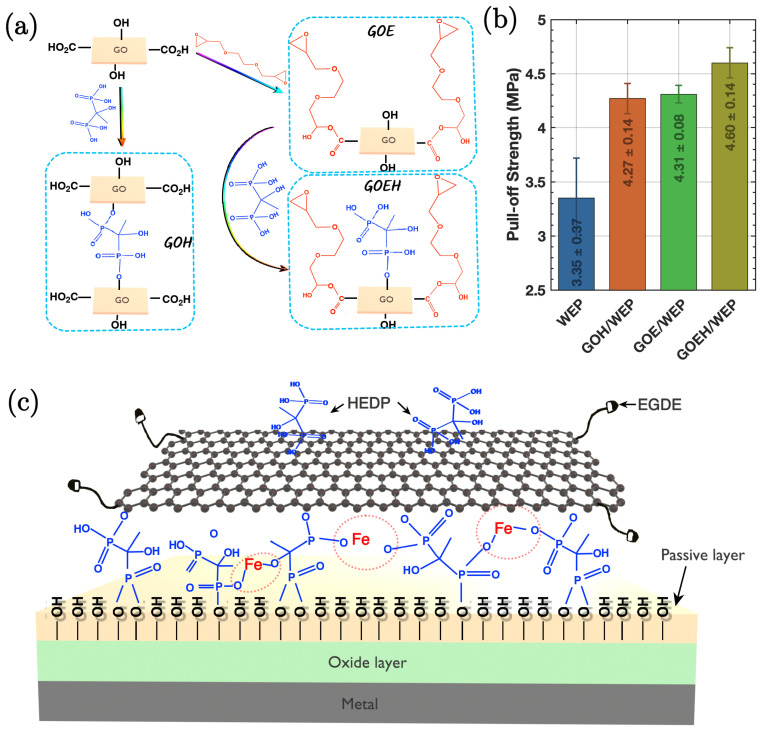
(**a**) Schematic illustration of the preparation process for GOH, GOE, and GOEH. (**b**) Adhesion strength of the specimens measured by pull-off testing. (**c**) Illustration of the corrosion protection mechanism for GOEH/WEP composite coatings [81].

Graphene, renowned for its exceptional mechanical and electrical properties, serves as an effective nanofiller in various polymer matrices, greatly enhancing their barrier properties against corrosion. Functionalised graphene and its derivatives when incorporated as nanofillers into polymeric coatings improve corrosion resistance. For instance, Yang et al. [81] demonstrated enhanced corrosion protection of waterborne epoxy (WEP) coatings when impregnated with functionalised graphene oxide derivatives, specifically GOEH. Figure 12a illustrates the syntheses of GOH, GOE, and GOEH. Incorporation of GOEH into WEP coatings provides a mechanism of both physical and chemical protective effects. The physical barrier is formed by well-dispersed graphene layers, which inhibit the diffusion of corrosive molecules/ions. The chemically mediated protection is primarily attributed to the presence of P-OH groups on GOEH, which can chelate with metal ions at the anode, forming insoluble metal–organic complexes. This reaction not only blocks the migration of Fe^2+^ ions but also plugs defects in the coating. Additionally, the P-OH groups enhance adhesion (as demonstrated through pull-off test results in Figure 12b), which is caused by reaction with the hydroxylated metal surface. This further retards the penetration of corrosive molecules along the GOEH/WEP composite interface.

The structural analysis of GOH, GOE, and GOEH reveals significant insights into their functional properties. FTIR and XPS spectroscopy indicate the successful grafting of HEDP onto GO, with characteristic peaks confirming the presence of P-O and P-OH groups. The reaction between the epoxide groups of EGDE and the carboxyl groups on GO results in the formation of GOE, which enlarges the interlayer spacing and improves compatibility with the WEP matrix, since structural modification is crucial for maintaining the dispersion of graphene layers, which is essential for the formation of an effective physical barrier.

Figure 12c illustrates the corrosion protection mechanism for GOEH/WEP composite coatings. The presence of EGDE molecules on GOEH allows for covalent bonding with the WEP matrix. This bonding is vital for preventing the penetration of corrosive molecules and ensuring the integrity of the coating. The structural characteristics of GOEH, such as the retention of P-OH groups, play a significant role in its ability to form strong bonds with metal substrates, contributing to the overall corrosion protection mechanism.

The performance of the GOEH/WEP coatings was evaluated through their impedance modulus, which stayed > 1.5 × 10^9^ Ω cm^2^ for five months with the addition of 0.5 wt% graphene derivatives. The sustenance of high impedance indicates excellent barrier properties and long-term stability of the coatings. The formation of a dense passivation layer by GOEH layers and their bonding with Fe^2^⁺ and hydroxyl groups on metal substrates further enhances the corrosion protection performance. This passivation layer acts as an additional barrier, preventing the ingress of corrosive species and maintaining the coating’s protective capabilities over time.

This review assessed the development of advanced corrosion protection coatings comprising graphene and its derivatives. The protection offered by such coatings can be leveraged to design protection systems with enhanced durability and performance in various industrial applications. Future research could explore the optimisation of graphene derivative concentrations and the potential for scaling up the production of these coatings for commercial use. Additionally, investigating the long-term environmental impact and sustainability of these coatings could provide valuable insights for their widespread adoption.

## 5. Conclusions

This review highlights the significant progress in utilising graphene-based composites, specifically graphene oxide (GO) and reduced graphene oxide (rGO), to enhance corrosion resistance in polymer matrices. Recent studies demonstrate that these composites provide superior barrier properties by extending ion diffusion paths and reducing corrosive agent permeability. Notably, some graphene-based polymer composites achieve an impressive corrosion protection efficiency of approximately 99% and a two- to four-order reduction in corrosion current density, underscoring the effectiveness of these materials in high-performance coatings. Additionally, advancements in functionalisation techniques have optimised GO dispersion within polymer matrices, enhancing compatibility, adhesion, and mechanical resilience.

However, despite these promising advancements, several key challenges remain that hinder the widespread practical application of graphene-based coatings. Issues such as the effective dispersion of graphene, achieving scalable production methods, and ensuring long-term environmental stability need to be addressed. For a comprehensive discussion of these challenges, please refer to Section 6.

Addressing these challenges will require targeted research efforts focusing on the development of scalable and cost-effective production techniques, innovating advanced functionalisation methods to maintain graphene’s inherent properties, and adopting sustainable and eco-friendly synthesis approaches to minimise environmental impacts.

These developments address the growing demand for sustainable, cost-efficient materials in industry by combining long-term durability with a reduced environmental impact. In summary, while graphene-impregnated coatings hold immense promise for enhancing corrosion resistance in a wide range of industrial applications, addressing the existing challenges through innovative research and development is imperative for their successful implementation. As research progresses, graphene-based polymer composites are emerging as promising candidates for next-generation anticorrosion technologies, offering a balance between high protective performance and ecological and economic sustainability.

## 6. Challenges and Future Perspectives

Despite the significant advancements in graphene-based anticorrosion coatings, several challenges remain that need to be addressed for their widespread adoption.

*Agglomeration of Graphene Sheets:* One of the primary challenges is the tendency of graphene and its derivatives, such as graphene oxide (GO) and reduced graphene oxide (rGO), to agglomerate within polymer matrices. This agglomeration disrupts uniform dispersion, leading to compromised barrier properties and reduced corrosion protection. Effective dispersion techniques, including covalent and non-covalent functionalisation, as well as the use of surfactants, are essential to overcome this issue.

*Functionalisation Challenges:* Achieving effective functionalisation of graphene without compromising its intrinsic electrical and barrier properties remains a significant challenge. Advanced functionalisation methods, such as plasma treatment, silanisation, and the incorporation of nanoparticles, could enhance compatibility with polymer matrices and improve corrosion resistance.

*Scalability and Cost:* The scalable production of high-quality graphene with minimal defects is both technically challenging and cost-prohibitive. Techniques such as chemical vapour deposition (CVD) and electrochemical exfoliation, although capable of producing high-quality graphene, are still expensive for large-scale applications. Research into cost-effective and scalable production methods, such as liquid-phase exfoliation and microwave-assisted reduction, is essential.

*Long-term Stability:* The long-term stability of graphene-impregnated polymeric coatings under harsh environmental conditions, including exposure to UV radiation, high salinity, and fluctuating pH levels, needs further investigation. The development of hybrid composites with synergistic properties offers a promising solution to this challenge.

## Figures and Tables

**Table 1:** Comparative efficiency of various polymer coatings with graphene nanosheets (GNSs).

Base Polymer	GNS Type	GNS Concentration	Fabrication Method	Observed Effect	Reference
Epoxy Resin	Graphene Oxide (GO)	0.5 wt%	Solution Mixing	Enhanced corrosion resistance and mechanical properties	[66]
Polyurethane	Functionalised GO	1.0 wt%	In situ Polymerisation	Improved barrier properties and thermal stability	[68]
Polyaniline	Reduced GO (rGO)	2.0 wt%	Electrophoretic Deposition	Increased electrical conductivity and anticorrosion performance	[67]
Polyvinyl Alcohol	GO-Silver Nanocomposite	0.3 wt%	Layer-by-Layer Assembly	Superior antibacterial activity and corrosion protection	[85]
Poly (methyl methacrylate)	GO	0.75 wt%	Melt Blending	Enhanced UV resistance and mechanical strength	[71]

**Table 2:** Corrosion resistance performance of graphene-integrated polymer composites.

Material Composition	Corrosive Medium	Inhibition Efficiency (%)	Tafel Slope (*βa/βc* in mV/Decade)	Corrosion Current Density (*I_corr_*, µA/cm^2^)	Corrosion Potential (*E_corr_*, V)	Ref.
PANI/NFGO	3.5% NaCl	93.5	95/110	0.12	−0.25	[86]
GO/Epoxy	3.5% NaCl	90.2	120/105	0.18	−0.3	[16]
rGO/Polypyrrole	1 M HCl	85.4	130/115	0.25	−0.35	[77]
Graphene/Polyurethane	3.5% NaCl	88.7	100/98	0.2	−0.28	[68]
GO/Chitosan	0.5 M NaCl	82.3	115/125	0.3	−0.32	[25]

## Data Availability

All data are available within the manuscript.
